# Prediction model for pulmonary infection risk in patients with cerebral hemorrhage and optimization of nursing intervention strategies

**DOI:** 10.3389/fmed.2026.1782100

**Published:** 2026-03-06

**Authors:** Lan Wang, Xiaoyan Ye, Zhaofeng Chen, Lei Zhang, Hai Zhou, Xin Wang, Qingqing Gao

**Affiliations:** Binhai County People's Hospital, Yancheng, Jiangsu Province, China

**Keywords:** cerebral hemorrhage, nursing intervention, prediction model, prognosis, pulmonary infection

## Abstract

**Objective:**

The incidence of pulmonary infections in patients with cerebral hemorrhage has significantly risen, profoundly impacting recovery and survival rates. This study aims to develop a predictive model for pulmonary infections in these patients and optimize nursing intervention strategies.

**Methods:**

A retrospective cohort design was employed, including hospitalized patients diagnosed with cerebral hemorrhage. Univariate logistic regression analysis identified risk factors for pulmonary infection, selecting indicators with statistical significance. Lasso regression and the Boruta algorithm were applied for variable selection optimization, followed by multivariate logistic regression to further refine the selection. Finally, a nomogram was constructed to predict pulmonary infection risk during hospitalization in these patients.

**Results:**

A total of 350 patients with cerebral hemorrhage meeting the inclusion criteria were enrolled in this study, with a pulmonary infection incidence of 49.1%. Significant risk factors included elevated C-reactive protein (CRP) levels (OR = 1.034, 95% CI: 1.018–1.050, *p* < 0.001), prolonged ICU stay (OR = 2.683, 95% CI: 2.077–3.465, *p* < 0.001), and four times daily oral care (OR = 0.199, 95% CI: 0.064–0.623, *p* = 0.006). The final model incorporated four key variables: proton pump inhibitor (PPI) use, CRP levels, oral care frequency, and intensive care unit (ICU) stay duration. The receiver operating characteristic (ROC) curve revealed an area under the curve (AUC) of 0.938.

**Conclusion:**

The development of an effective predictive model for pulmonary infections in patients with cerebral hemorrhage enhances clinicians’ ability to accurately identify high-risk patients, supporting improved clinical decision-making. Integrating this model into clinical practice, alongside targeted nursing interventions, can reduce the incidence of pulmonary infections and improve overall patient prognosis.

## 1 Introduction

Cerebral hemorrhage is a severe neurological disorder (1), with pulmonary infection being a common complication affecting 16 to 68% of patients (2, 3). In the neurosurgical intensive care unit (ICU), the risk is further exacerbated by factors such as impaired consciousness, prolonged bed rest, and the necessity of tracheal intubation (4). Pulmonary infections not only impede neurological rehabilitation but also trigger a vicious cycle of systemic inflammation, hypoxemia, and elevated intracranial pressure (5–7) leading to prolonged hospital stays, increased medical expenses, and heightened mortality risk (8).

The occurrence of pulmonary infection in these patients is influenced by multiple physiological and clinical factors. Proton pump inhibitors (PPIs), while essential for gastric protection, have been found to alter the intestinal microbiota and gastric pH, potentially increasing infection risk (9). Similarly, C-reactive protein (CRP) serves as a critical marker, where elevated levels often correlate with post-stroke immunosuppression and a higher susceptibility to pulmonary complications (10).

Clinical procedures and nursing interventions play a pivotal role in modulating this risk. While procedures like tracheal intubation and nasogastric feeding are often indispensable despite their link to infection (11–13), evidence-based nursing measures—such as thorough oral care, timely repositioning, and nutritional support—have demonstrated significant potential in reducing infection rates (14). However, many existing predictive models rely heavily on immutable factors (e.g., age or hemorrhage location), often overlooking the impact of modifiable nursing protocols.

Therefore, early identification of high-risk patients through an effective predictive tool is essential for timely intervention. This study aims to develop and validate a nomogram that integrates both clinical indicators and modifiable nursing parameters, such as oral care frequency. By providing a reliable risk stratification tool, we seek to assist clinicians in implementing targeted preventive measures and ultimately improving patient outcomes.

## 2 Methods

### 2.1 Research population

This study was conducted at Binhai County People’s Hospital from January 2023 to June 2025. A retrospective cohort design was employed, with 350 inpatients diagnosed with cerebral hemorrhage based on clinical features and imaging examinations. The study adhered to the ethical guidelines of the Helsinki Declaration (revised in 2013) and received approval from the Ethics Committee of Binhai County People’s Hospital (Approval Number: 2025-BYKYLL-031). Written informed consent was obtained from all patients or their legal guardians. Inclusion criteria were as follows: (1) Age ≥ 18 years; (2) Clinical diagnosis of cerebral hemorrhage; (3) Admission to the neurosurgery department or ICU; (4) Availability of complete clinical data. Exclusion criteria were as follows: (1) Missing data on key laboratory indicators; (2) Refusal to participate or inability to provide informed consent.

### 2.2 Collection of clinical data and follow-up

Data were collected through the hospital’s electronic medical record system, including: (1) Demographic characteristics: age, gender, body mass index (BMI), smoking history, and alcohol consumption; (2) Laboratory indicators: white blood cell count (WBC), hemoglobin (Hb), platelet count (PLT), serum albumin, and CRP levels; (3) Treatment measures: mechanical ventilation, tracheotomy, nasogastric tube insertion, deep vein catheterization, PPI use, and sedation and analgesia; (4) Clinical characteristics: cause of cerebral hemorrhage (traumatic/spontaneous), frequency of oral care (2, 3, or 4 times/day), frequency of suctioning (2, 3, or 4 times/day), and ICU stay duration. All laboratory tests were conducted using a standardized automatic analysis system, with quality control performed by laboratory physicians.

### 2.3 Outcome

The primary outcome measure was pulmonary infection that occurred in patients with cerebral hemorrhage during their hospital stay. The definition was based on the “Chinese Guidelines for Diagnosis and Treatment of Hospital-acquired pulmonary infection and Ventilator-associated pulmonary infection (2018 Edition)”. To ensure diagnostic consistency and minimize monitoring bias, two senior clinicians independently reviewed the electronic medical records, imaging examinations, and laboratory test results. In case of disagreement, a third senior consultant was consulted to reach a consensus. The diagnostic criteria for pulmonary infection include: (1) clinical symptoms (fever, purulent sputum, etc.); (2) new infiltrates found on imaging examinations; (3) positive microbiological culture; (4) elevated inflammatory markers (WBC > 10*10^9^ /L or CRP > 50 mg/L), and at least two of these criteria must be met for a definitive diagnosis. Since patients with severe neurological diseases share overlapping clinical manifestations and their prevention measures are also relatively consistent, hospital-acquired pulmonary infection, ventilator-associated pulmonary infection, and aspiration pulmonary infection are all classified as pulmonary infections during hospitalization to ensure the wide applicability and statistical reliability of this model in clinical practice. To ensure a clear temporal sequence between predictors and outcomes, all predictive variables were recorded prior to the onset of infection. Specifically, nursing interventions such as oral care frequency were calculated as the mean daily frequency from admission until the day of infection diagnosis. The timing of infection was adjudicated as the date of the first appearance of new or progressive infiltrates on imaging, as determined by two independent clinicians blinded to the predictor data. This procedure ensures the reliability of the outcome adjudication and the predictive validity of the model.

### 2.4 Statistical analysis

#### 2.4.1 Descriptive statistics and univariate analysis

Baseline characteristics of the included patients with cerebral hemorrhage were summarized. Continuous variables were tested for normality using the Shapiro–Wilk test. Variables with non-normal distribution were expressed as median (interquartile range) and compared between groups using the Wilcoxon rank-sum test. Categorical variables were expressed as frequency (percentage) and compared using the chi-square test. Univariate logistic regression was performed to identify potential risk factors for pulmonary infection.

#### 2.4.2 Variable selection for multivariate modeling

Variables with a *p*-value < 0.05 from the univariate analysis were selected for further feature selection to address multicollinearity and optimize the predictor set. Two complementary methods were used: (1) Lasso regression with 10-fold cross-validation, where the penalty parameter (*λ*) was determined by minimizing the mean squared error. (2) The Boruta algorithm, a wrapper method based on random forest, was executed for 500 iterations to assess variable importance by comparing original variables with shadow variables.

Variables consistently identified as significant by both methods were retained for the final model.

#### 2.4.3 Model building and evaluation

A multivariate logistic regression model was constructed using the selected variables. A nomogram was developed based on this model to predict the risk of in-hospital pulmonary infection after cerebral hemorrhage. The model’s discriminative ability was assessed using the receiver operating characteristic (ROC) curve and the area under the curve (AUC). To assess the internal validity and minimize overfitting, internal validation was performed using a bootstrapping method with 1,000 resamples to calculate the optimism-corrected C-index. Model fit was evaluated with a calibration curve and the Hosmer-Lemeshow goodness-of-fit test. The point allocation in the nomogram was derived from the raw regression coefficients (*β*) of the logistic model. The visual length of each axis is determined by the product of the coefficient and the variable’s potential clinical range. Consequently, while a categorical variable (e.g., ICU length of stay) may have a larger odds ratio (OR), a continuous variable with a wide distribution range (e.g., CRP) may occupy a longer visual axis, reflecting its cumulative contribution across its entire clinical spectrum. To evaluate the clinical utility and net benefit of the model, Decision Curve Analysis (DCA) was conducted by calculating the net benefits at different threshold probabilities. Furthermore, an optimal clinical decision threshold was determined based on the maximum net benefit and the Youden index to provide evidence-based guidance for bedside clinical interventions.

All analyses were performed using R software (version 4.3.0) and STATA 17.0 (64-bit). A two-sided *p*-value < 0.05 was considered statistically significant.

## 3 Results

### 3.1 Demographic and clinical characteristics of patients with cerebral hemorrhage

A total of 350 patients with cerebral hemorrhage who met the inclusion criteria were included in this study. Among them, 172 cases (49.1%) developed pulmonary infection, while 178 cases (50.9%) did not. As shown in Table 1, no significant statistical differences were observed in demographic characteristics (age, gender, BMI, smoking, and drinking history) between the pulmonary infection and non-infection groups (all *p* > 0.05). However, inflammatory markers in the infection group were significantly elevated, including CRP (19.86 vs. 10.00, *p* < 0.001) and WBC count (13.80 vs. 12.57, *p* = 0.054). Additionally, the Glasgow Coma Scale (GCS) score was lower in the infection group (8 vs. 11, *p* < 0.001), indicating more severe consciousness impairment. In terms of treatment, patients in the pulmonary infection group were more frequently subjected to mechanical ventilation (68.0% vs. 39.9%, *p* < 0.001), tracheotomy (34.3% vs. 5.1%, *p* < 0.001), and analgesia and sedation (57.6% vs. 27.5%, *p* < 0.001). The duration of mechanical ventilation (37.5 vs. 0 h) and analgesia (4.5 vs. 0 h) was significantly prolonged (all *p* < 0.001). Concerning comorbidities, patients with chronic obstructive pulmonary disease (COPD) had a higher risk of infection (30.2% vs. 19.1%, *p* = 0.016). The length of ICU stay was significantly longer in the infection group (9 vs. 4 days, *p* < 0.001). Differences in the frequency of oral care (2 times/day: 27.9% vs. 17.4%) and sputum suction (3 times/day: 54.7% vs. 39.9%) were also statistically significant (all *p* < 0.05). It is worth noting that the overall mortality rate of this hospital was 20.6% (72/350). The mortality rate in the pulmonary infection group was significantly higher than that in the non-infection group (31.4% vs. 10.1%, *p* < 0.001). Regarding the causes of death, the proportion of non-neurological deaths (such as respiratory failure and sepsis) in the pulmonary infection group was significantly higher than that in the non-infection group (35.2% vs. 11.1%, *p* = 0.002), among which neurological factors (such as brain herniation) remained the main cause of death.

**Table 1:** Baseline characteristics of the included population.

Variables	Total (*N* = 350)	non-pulmonary infection (*N* = 178)	Pulmonary infection (*N* = 172)	*p*
Demographic characteristics				
Age, years	67.00(57.00,74.00)	66.00(56.00, 74.00)	67.00(58.00, 75.00)	0.414
Sex, n (%)				0.292
Female	109 (31.1%)	60 (33.7%)	49 (28.5%)	
Male	241 (68.9%)	118 (66.3%)	123 (71.5%)	
BMI (kg/m^2^)	24.22(22.49,27.04)	23.88(22.02, 26.83)	24.22(22.64, 27.08)	0.091
Smoking, n (%)				0.120
No	265 (75.7%)	141 (79.2%)	124 (72.1%)	
Yes	85 (24.3%)	37 (20.8%)	48 (27.9%)	
Alcohol, n (%)				0.466
No	213 (60.9%)	105 (59.0%)	108 (62.8%)	
Yes	137 (39.1%)	73 (41.0%)	64 (37.2%)	
Clinical features				
WBC	13.42(9.84,16.95)	12.57(9.64, 16.34)	13.80(10.22, 17.46)	0.054
Hb	125.00(111.00,136.00)	124.00(113.00, 136.00)	126.00(110.25, 136.75)	0.987
PLT	158.00(121.00,203.50)	158.00(117.75, 199.50)	161.50(123.00, 209.75)	0.294
CRP	10.00(0.80,36.60)	10.00(0.80, 15.43)	19.86(10.00, 58.52)	<0.001
Albumin	41.00(38.77,43.42)	41.15(39.10, 43.42)	40.85(38.20, 43.55)	0.368
GCS	10.00(6.00,12.00)	11.00(7.00, 13.00)	8.00(5.00, 12.00)	<0.001
Operative treatment				
Ventilation, n (%)				<0.001
No	162 (46.3%)	107 (60.1%)	55 (32.0%)	
Yes	188 (53.7%)	71 (39.9%)	117 (68.0%)	
Ventilation_Time (h)	2.00(0.00,76.25)	0.00(0.00, 16.50)	37.50(0.00, 144.00)	<0.001
Tracheotomy, n (%)				<0.001
No	282 (80.6%)	169 (94.9%)	113 (65.7%)	
Yes	68 (19.4%)	9 (5.1%)	59 (34.3%)	
Pain_Relief, n (%)				<0.001
No	202 (57.7%)	129 (72.5%)	73 (42.4%)	
Yes	148 (42.3%)	49 (27.5%)	99 (57.6%)	
Pain_Relief_Time (h)	0.00(0.00,31.02)	0.00(0.00, 3.00)	4.50(0.00, 63.87)	<0.001
Nasogastric_Tube, n (%)				<0.001
No	110 (31.4%)	71 (39.9%)	39 (22.7%)	
Yes	240 (68.6%)	107 (60.1%)	133 (77.3%)	
Nasogastric_tube_Time (d)	4.00(0.00,7.00)	3.00(0.00, 4.00)	6.00(1.00, 12.00)	<0.001
Venous_Catheterization, n (%)				<0.001
No	196 (56.0%)	119 (66.9%)	77 (44.8%)	
Yes	154 (44.0%)	59 (33.1%)	95 (55.2%)	
Venous_Catheterization_Time (d)	0.00(0.00,6.00)	0.00(0.00, 3.00)	3.00(0.00, 9.75)	<0.001
PPI, n (%)				<0.001
No	184 (52.6%)	112 (62.9%)	72 (41.9%)	
Yes	166 (47.4%)	66 (37.1%)	100 (58.1%)	
Accompanying the disease				
Diabetes, n (%)				0.002
No	225 (64.3%)	128 (71.9%)	97 (56.4%)	
Yes	125 (35.7%)	50 (28.1%)	75 (43.6%)	
Hypertension, n (%)				0.989
No	240 (68.6%)	122 (68.5%)	118 (68.6%)	
Yes	110 (31.4%)	56 (31.5%)	54 (31.4%)	
COPD, n (%)				0.016
No	264 (75.4%)	144 (80.9%)	120 (69.8%)	
Yes	86 (24.6%)	34 (19.1%)	52 (30.2%)	
Other characteristics				
Type_of_damage, n (%)				0.246
Traumatic bleeding	94 (26.9%)	43 (24.2%)	51 (29.7%)	
Spontaneous bleeding	256 (73.1%)	135 (75.8%)	121 (70.3%)	
Frequency_Oral_Care, n (%)				<0.001
2 times a day	79 (22.6%)	31 (17.4%)	48 (27.9%)	
3 times a day	170 (48.6%)	80 (44.9%)	90 (52.3%)	
4 times a day	101 (28.9%)	67 (37.6%)	34 (19.8%)	
Frequency_suctioning, n (%)				0.003
2 times a day	86 (24.6%)	43 (24.2%)	43 (25.0%)	
3 times a day	165 (47.1%)	71 (39.9%)	94 (54.7%)	
4 times a day	99 (28.3%)	64 (36.0%)	35 (20.3%)	
Raise_Bed, n (%)				0.033
<30°	169 (48.3%)	76 (42.7%)	93 (54.1%)	
≥30°	181 (51.7%)	102 (57.3%)	79 (45.9%)	
ICU_Time (d)	5.00(4.00,9.00)	4.00(3.00, 5.00)	9.00(6.25, 13.75)	<0.001
Outcome				
Hospitalization mortality rate, n (%)	72 (20.6%)	18 (10.1%)	54 (31.4%)	<0.001
Classification of causes of death, n (%)				0.002
Cause of death of the nervous system	51 (70.8%)	16 (88.9%)	35 (64.8%)	
Non-neurological causes of death	21 (29.2%)	2 (11.1%)	19 (35.2%)	

### 3.2 Results of univariate analysis related to pulmonary infection

Univariate logistic regression analysis (Table 2) revealed 23 variables significantly associated with the risk of pulmonary infection in patients with cerebral hemorrhage (*p* < 0.05). These variables included: lower GCS score, multiple invasive procedures (mechanical ventilation, tracheotomy, analgesia and sedation, nasogastric tube insertion, deep vein catheterization), their duration, laboratory indicators (elevated CRP), comorbidities (diabetes, COPD), drug use (PPI), and prolonged ICU stay. Protective factors included four times daily oral care and elevating the bed head by ≥ 30°. All variables with *p* < 0.05 were included in the subsequent variable selection process.

**Table 2:** Univariate and multivariate logistic regression analyze of pulmonary infection in patients with cerebral hemorrhage.

Variables	Univariate analysis	*p*-value	Multivariate analysis	*p*-value
	OR(95%CI)		OR(95%CI)	
Age,years	1.010(0.995,1.025)	0.190		
BMI (kg/m2)	1.010(0.987,1.034)	0.399		
GCS	0.874(0.827,0.925)	<0.001	0.910(0.808,1.024)	0.118
Ventilation_Time (h)	1.013(1.008,1.017)	<0.001	0.999(0.991,1.007)	0.866
Pain_Relief_Time (h)	1.012(1.006,1.018)	<0.001		
Nasogastric_tube_Time (d)	1.256(1.179,1.338)	<0.001		
Venous_Catheterization_Time (d)	1.206(1.134,1.283)	<0.001		
WBC	1.041(1.001,1.082)	0.044		
Hb	0.999(0.989,1.009)	0.828		
PLT	1.002(0.999,1.006)	0.174		
CRP	1.026(1.016,1.035)	<0.001	1.034(1.018,1.050)	<0.001
Albumin	0.954(0.908,1.002)	0.060		
ICU_Time	2.356(1.944,2.855)	<0.001	2.683(2.077,3.465)	<0.001
Sex, n (%)				
Female	Reference			
Male	1.276(0.811,2.010)	0.292		
Smoking, n (%)				
No	Reference			
Yes	1.475(0.902,2.413)	0.121		
Alcohol, n (%)				
No	Reference			
Yes	0.852(0.555,1.310)	0.466		
Diabetes, n (%)				
No	Reference		Reference	
Yes	1.979(1.269,3.088)	0.003	1.658(0.729,3.769)	0.228
Hypertension, n (%)				
No	Reference			
Yes	0.997(0.635,1.566)	0.989		
COPD, n (%)				
No	Reference			
Yes	1.835(1.118,3.012)	0.016		
Type_of_damage				
Traumatic bleeding	Reference			
Spontaneous bleeding	0.756(0.470,1.214)	0.247		
Ventilation				
No	Reference			
Yes	3.206(2.067,4.973)	<0.001		
Tracheotomy				
No	Reference			
Yes	2.804(1.674,4.564)	<0.001		
Pain_Relief				
No	Reference		Reference	
Yes	3.570(2.284,5.581)	<0.001	1.178(0.479,2.896)	0.721
Nasogastric_Tube				
No	Reference			
Yes	2.263(1.420,3.607)	<0.001		
Venous_Catheterization				
No	Reference			
Yes	2.488(1.613,3.838)	<0.001		
PPI				
No	Reference		Reference	
Yes	2.357(1.534,3.621)	<0.001	2.133(0.977,4.658)	0.057
Frequency_Oral_Care, n (%)				
2 times a day	Reference		Reference	
3 times a day	0.727(0.422,1.250)	0.249	0.613(0.238,1.581)	0.311
4 times a day	0.328(0.178,0.604)	<0.001	0.199(0.064,0.623)	0.006
Frequency_suctioning, n (%)				
2 times a day	Reference		Reference	
3 times a day	1.324(0.785,2.234)	0.293	2.143(0.847,5.424)	0.108
4 times a day	0.547(0.303,0.987)	0.045	0.359(0.117,1.096)	0.072
Raise_Bed, n (%)				
<30°	Reference		Reference	
≥30°	0.633(0.415,0.965)	0.034	0.538(0.247,1.174)	0.119

### 3.3 Variable selection results

For the prediction model, single-factor logistic regression, LASSO regression, and the Boruta algorithm were employed for variable selection.

#### 3.3.1 LASSO regression selection

**Figure 1:**
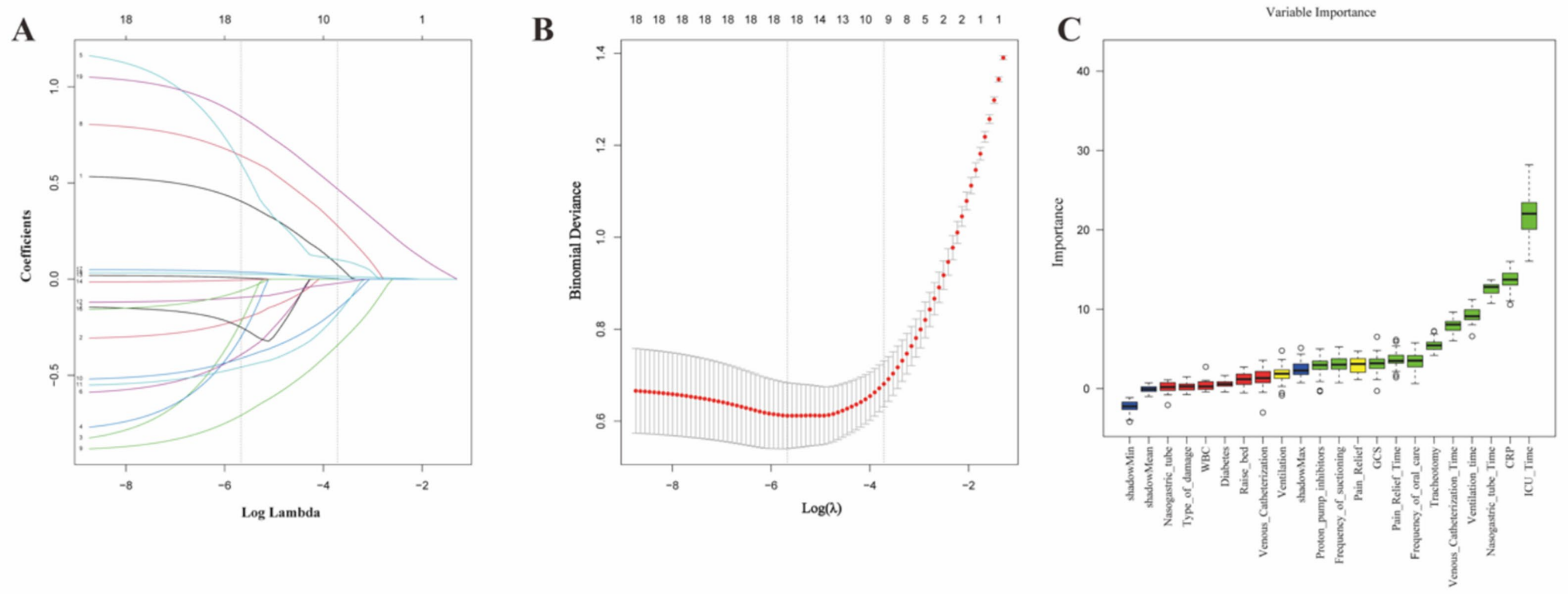
Identification and selection of optimal predictive features. **(A,B)** LASSO regression analysis: The least absolute shrinkage and selection operator (LASSO) method was employed to minimize overfitting and identify the most stable predictors by penalizing less relevant variables. **(C)** Boruta feature selection: This shadow-feature-based algorithm was used to confirm the “all-relevant” predictors.

LASSO regression analysis (Figures 1A,B) showed that when *λ* was set to the value corresponding to the minimum mean square error (λ = 0.003), the model retained 19 variables. When λ was set to the minimum value within one standard error (λ = 0.024), the model was further simplified to 10 variables, including diabetes, analgesia and sedation, PPI use, frequency of oral care, frequency of suctioning, head elevation angle, GCS score, WBC count, CRP, and ICU hospitalization duration.

#### 3.3.2 Boruta algorithm screening

The Boruta feature importance assessment (Figure 1C) identified several key variables: GCS score, mechanical ventilation duration, tracheotomy, analgesia duration, nasogastric tube retention duration, deep vein catheterization duration, PPI use, CRP, ICU stay duration, oral care frequency, and suction frequency. Conversely, diabetes, bleeding cause, nasogastric tube use, deep vein catheterization, WBC count, and head-of-bed elevation angle were excluded. Mechanical ventilation and analgesia/sedation were categorized as “tentative” variables.

Following a comprehensive evaluation of the Lasso regression and Boruta results, the final model incorporated 10 variables, encompassing clinical interventions (e.g., mechanical ventilation, analgesia, sedation, nasogastric tube, PPI use), laboratory indicators (e.g., CRP), and patient-specific conditions (e.g., GCS, ICU duration), establishing a robust and precise prediction framework.

### 3.4 Independent risk factors for pulmonary infection in patients with cerebral hemorrhage

A multivariate logistic regression analysis, based on the selected variables, identified independent risk factors (Table 2). The findings revealed that, after adjusting for other factors, elevated CRP levels (OR = 1.034, 95% CI: 1.018–1.050, *p* < 0.001) and prolonged ICU stays (OR = 2.683, 95% CI: 2.077–3.465, *p* < 0.001) were independent risk factors for pulmonary infection in patients with cerebral hemorrhage. Performing four oral care sessions daily (OR = 0.199, 95% CI: 0.064–0.623, *p* = 0.006) emerged as an independent protective factor. While PPI use was associated with an increased infection risk, it did not reach statistical significance (OR = 2.133, 95% CI: 0.977–4.658, *p* = 0.057).

### 3.5 Construction of a predictive model for pulmonary infection in patients with cerebral hemorrhage and evaluation of model efficacy

**Figure 2:**
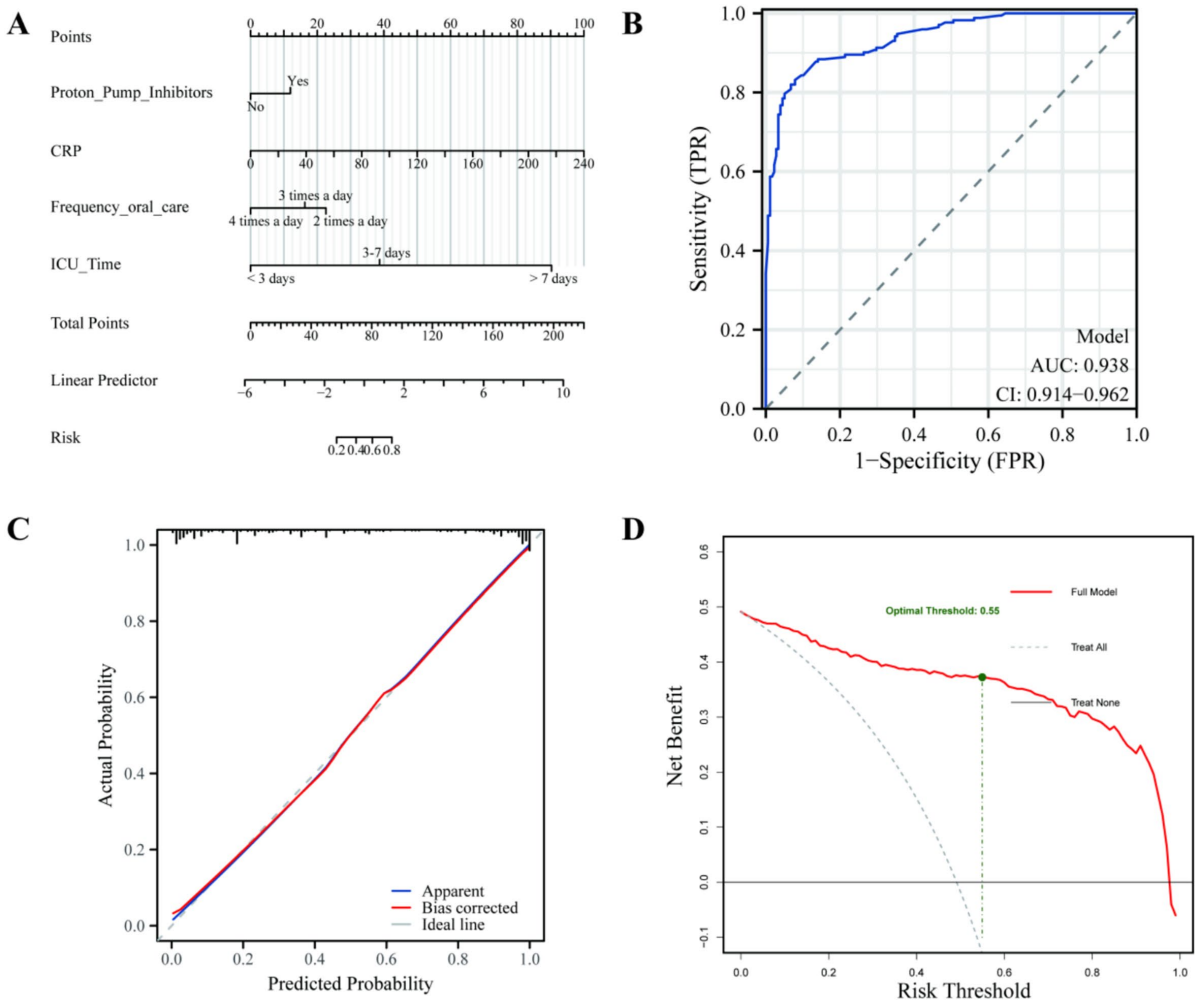
Development and performance evaluation of the pulmonary infection risk nomogram. **(A)** Nomogram: A visual tool for individualized risk estimation based on the identified predictors. **(B)** Receiver operating characteristic (ROC) curve: Demonstrates the model’s discriminative ability, with a high AUC (0.938) indicating excellent performance in distinguishing between infected and non-infected patients. **(C)** Calibration curve: Shows the consistency between the predicted probability and the observed outcomes. **(D)** Decision curve analysis (DCA): Evaluates the clinical utility by calculating the net benefit.

This study developed an independent risk factor prediction model to identify pulmonary infection in patients with cerebral hemorrhage during hospitalization and thoroughly evaluated its efficacy. The final model included four key variables: PPI use, CRP, frequency of oral care, and ICU hospitalization duration, which served as the main inputs. Figure 2A illustrates the risk scores of each variable and their contributions to the overall risk. By calculating the linear combination of these variables and converting them into probabilities, the model effectively predicts the risk of pulmonary infection in patients with cerebral hemorrhage. The model’s efficacy was comprehensively assessed. First, the overall significance was tested using the likelihood ratio test, which yielded a chi-square value of 268.06 and a *p*-value < 0.05, confirming the model’s significance. The model’s discrimination ability was then evaluated using the C-index, which resulted in a value of 0.938 (95% CI: 0.914–0.962), significantly exceeding the threshold of 0.9, indicating high accuracy in distinguishing whether patients with cerebral hemorrhage would develop pulmonary infection. Internal validation using a bootstrapping method with 1,000 resamples yielded an optimism-corrected C-index of 0.915, demonstrating the model’s robust predictive stability. For calibration, the Hosmer-Lemeshow Goodness of Fit test showed a chi-square value of 7.167 with a *p*-value of 0.5187, indicating no significant difference between the predicted and observed values, and confirming good model calibration. The ROC curve demonstrated an AUC of 0.938, further supporting the model’s strong discriminatory power (Figure 2B). The calibration curve showed that the actual probability closely matched the predicted probability, and after bias correction, the results remained close to the ideal line, further validating the model’s high accuracy and reliability (Figure 2C). Finally, the DCA results showed that within the broad threshold range of 0.1 to 0.9, the net benefit of this comprehensive model (Figure 2D, red solid line) was significantly higher than that of the full intervention or no-intervention strategies, demonstrating its excellent clinical practicability. Through analysis, the optimal clinical decision threshold was determined to be 0.55. This means that when the model predicts a pulmonary infection risk of over 55%, taking targeted nursing preventive measures (such as enhancing oral care) can provide the maximum clinical net benefit for the patient. In conclusion, this predictive model not only exhibited excellent discrimination and calibration but also demonstrated high predictive value in practical applications. It represents an effective tool for assessing the risk of pulmonary infection in patients with cerebral hemorrhage.

## 4 Discussion

In this study, we successfully developed and validated a clinical nomogram with high predictive accuracy (AUC 0.938) for identifying pulmonary infection risk in patients with cerebral hemorrhage. The most relevant findings indicate that frequent oral care (4 times/day) and controlled ICU stay duration are critical modifiable protective and risk factors, respectively. Furthermore, our results emphasize the clinical gravity of these infections: patients in the pulmonary infection group faced a significantly higher hospital mortality rate (31.4% vs. 10.1%, *p* < 0.001), with non-neurological complications such as respiratory failure becoming a predominant cause of death. While the observed infection incidence of 49.1% aligns with the reported range of 21 to 56% in critically ill stroke populations (10, 15), our model provides a more granular tool for bedside risk stratification, moving beyond simple incidence reporting to actionable clinical intervention.

Recent studies have suggested a potential link between PPI use and the occurrence of pulmonary infections, particularly in patients with cerebral hemorrhage. PPIs significantly reduce gastric acid secretion by inhibiting the hydrogen-potassium ATPase in gastric wall cells. Given that patients with cerebral hemorrhage are at increased risk of aspiration due to factors such as altered consciousness and swallowing difficulties, PPI use may disrupt the stomach’s internal environment, fostering bacterial growth and spread, thereby heightening the risk of pulmonary infection (16, 17). Consistent with our findings, numerous studies have indicated a positive correlation between PPI use and the incidence of pulmonary infections (18). Additionally, PPIs may contribute to the risk of pulmonary infections by altering the gastric pH and disrupting the intestinal microbiota, leading to the proliferation of harmful bacteria (19).

This study demonstrates that elevated CRP levels (OR = 1.034, 95% CI: 1.018–1.050, *p* < 0.001) are an independent risk factor for pulmonary infection in patients with cerebral hemorrhage. CRP plays a pivotal regulatory role in inflammatory responses, particularly in pathological conditions such as infection, trauma, and autoimmune diseases, where its levels significantly increase (20). The primary function of CRP is to facilitate the clearance of pathogens and tissue repair by binding to ligands like phosphatidylcholine. CRP levels are closely associated with the risk of pulmonary infection. In patients with cerebral hemorrhage, elevated CRP often indicates a heightened risk of infection, particularly in ICU patients, where CRP is a key marker for assessing infection and inflammation (21). For instance, studies have shown that CRP levels are significantly higher in patients with pulmonary infection compared to non-infected patients, and the increase in CRP correlates with the severity of infection and prognosis (22). Moreover, CRP can serve not only for early infection detection but also as a marker for monitoring treatment efficacy, enabling timely adjustments to treatment plans (23). Therefore, integrating CRP into the predictive model for pulmonary infection risk in patients with cerebral hemorrhage during hospitalization holds significant clinical value.

Additionally, longer ICU stays increase the risk of pulmonary infection in patients with cerebral hemorrhage (24). Additionally, the duration of mechanical ventilation, sedative use frequency, and underlying conditions such as diabetes and chronic lung disease are important contributors to the risk of pulmonary infection (25, 26). For instance, a retrospective study found that patients with ICU stays exceeding 7 days had a significantly higher incidence of pulmonary infection compared to those with shorter stays. In the ICU, immune function is often compromised, and prolonged bed rest impedes effective lung drainage, further raising the risk of infection (12, 27). It should be noted that there may be a bidirectional relationship between the length of stay in the ICU and pulmonary infection. A prolonged hospitalization increases the chances of patients being exposed to hospital-acquired pathogens and undergoing invasive procedures, while a confirmed infection may also prolong the patient’s stay in the intensive care unit. In this model, the length of stay in the intensive care unit can serve as an exposure window and a comprehensive indicator of the severity of the patient’s overall condition. Therefore, reducing ICU stay duration, optimizing respiratory management, and enhancing infection prevention measures are critical to decreasing pulmonary infection rates and improving patient prognosis.

It is also important to consider the potential influence of brain topography on these outcomes. It is well-recognized that the topography and etiology of intracerebral hemorrhage (e.g., lobar vs. deep subcortical) lead to distinct clinical profiles and prognostic outcomes. As noted in recent studies, acute spontaneous lobar hemorrhages often involve non-hypertensive mechanisms and carry a more severe early prognosis compared to deep subcortical hemorrhages (28). However, in our univariate analysis, the type of brain damage (spontaneous vs. traumatic) did not show a statistically significant association with the incidence of pulmonary infection (*p* = 0.247). This suggests that in the neurosurgical ICU setting, the risk of respiratory complications is more heavily influenced by systemic factors such as impaired consciousness (GCS, *p* < 0.001), prolonged ICU exposure, and the quality of nursing care rather than the initial anatomical location of the hemorrhage.

Performing four oral care sessions daily (OR = 0.199, 95% CI: 0.064–0.623, *p* = 0.006) is an independent protective factor against pulmonary infections in patients with cerebral hemorrhage. Increasing oral care frequency to twice daily or more significantly reduces the incidence of pulmonary infections and improves overall patient health (29, 30). Oral care plays a critical role in critically ill patients, as the bacterial communities in the mouth are closely linked to pulmonary infections. Pathogenic bacteria from the oral cavity can enter the airways through inhalation or accidental aspiration, leading to pulmonary infections (31). Critically ill patients are often at higher risk due to issues such as swallowing difficulties and confusion, which facilitate bacterial accumulation in the mouth. Regular oral care to eliminate bacteria and biofilms can significantly reduce the incidence of HAP and ventilator-associated pulmonary infection (VAP) (32). Thus, implementing a systematic oral care protocol for critically ill patients is associated with a reduced risk of pulmonary infections and represents a potential strategy for prevention. Additionally, increasing the frequency of sputum suction and elevating the head of the bed can improve lung function, facilitate sputum clearance, and reduce infection risk (9). The application of advanced nursing technologies has shown notable benefits in enhancing care quality for patients with cerebral hemorrhage and reducing pulmonary infection risk. For example, nurse-led goal-oriented pulmonary physical therapy has been demonstrated to shorten mechanical ventilation duration, lower ICU mortality, and reduce 28-day mortality rates (33). The integration of such technologies not only improves care efficiency but also improves patient outcomes, offering new approaches and methods for future nursing practices. However, we must carefully interpret the protective effect of the four-times-daily oral care. This association may be influenced by the severity of the patient’s condition or the allocation of nursing resources, rather than being merely a direct causal relationship. For instance, patients with extremely unstable conditions or those requiring continuous life support interventions may postpone routine care, such as frequent oral cleaning, due to medical priorities. Therefore, a higher frequency of oral care may serve as an alternative indicator of clinical stability, or reflect a higher ratio of care to patients. However, in our multivariate analysis, after adjusting for physiological severity indicators such as C-reactive protein and length of stay in the intensive care unit, the independent protective effect of frequent oral care remained statistically significant. This indicates that, despite the presence of confounding factors, standardized and frequent oral cleaning remains a key component in reducing the risk of stroke-related pulmonary infection.

Compared with previously released tools (such as machine-learning models based on imaging), a notable feature of this model is its inclusion of modifiable clinical parameters. While existing models often rely on immutable factors like hemorrhage location or age, our study identified the frequency of oral care as a significant independent factor associated with lower infection risk (OR = 0.199). From a predictive perspective, this adds incremental value to the model’s utility as a decision-support tool. The high AUC (0.938) and the accuracy demonstrated by the calibration curve support its reliability in risk stratification. In terms of clinical interpretation, the nomogram’s structure reflects the distinct nature of these predictors. Despite the large effect size of ICU length of stay as a risk factor, its discrete nature as a categorical variable results in specific point increments in the nomogram. In contrast, CRP provides a continuous gradient of risk, allowing for more granular risk assessment at the bedside. This design ensures that the model captures both acute categorical risks and evolving physiological inflammatory states. While the observational nature of this study precludes a definitive causal claim, the inclusion of such modifiable factors allows clinicians to identify patients who might benefit from optimized nursing protocols. Rather than implying a guaranteed reduction in infection through increased care frequency alone, this model serves to highlight high-risk profiles where targeted, evidence-based nursing practices could be prioritized.

This study has several limitations that should be emphasized. First, the single-center, retrospective design may introduce selection and information biases, and the relatively small sample size limits the generalizability of our findings. Second, although we performed internal validation via bootstrapping, the model lacks robust external validation in independent cohorts. Third, the potential for reverse causality exists among key predictors; while we defined variables to mitigate this, factors like ICU stay duration can be both a risk factor and a consequence of subclinical changes. Similarly, the protective effect of oral care frequency may be confounded by unmeasured factors such as staffing levels.

Looking forward, several avenues for future research emerge. Future multi-center, prospective studies are required to externally validate the clinical utility of this nomogram. Additionally, incorporating advanced biological markers, such as immunological profiling or metagenomic data, could further refine the model’s precision. Most importantly, interventional clinical trials are warranted to determine whether proactive adjustments to nursing protocols—guided by our risk stratification tool—can directly reduce infection rates and improve patient survival. Finally, integrating this predictive tool into automated electronic health record (EHR) systems could provide real-time decision support for neurosurgical ICU teams.

## 5 Conclusion

In the treatment and care of patients with cerebral hemorrhage, the risk of pulmonary infection is a critical issue that requires attention. Optimizing treatment strategies and nursing plans necessitates a focus on both individual factors and the interrelationships among multiple factors. Future research should deepen the exploration of these complex interactions to provide more effective guidance for clinical practice, ultimately reducing the incidence of pulmonary infections and improving the quality of prognosis for patients with cerebral hemorrhage.

## Data Availability

The raw data supporting the conclusions of this article will be made available by the authors, without undue reservation.
